# ﻿A phylogeny of the Triraphideae including *Habrochloa* and *Nematopoa* (Poaceae, Chloridoideae)

**DOI:** 10.3897/phytokeys.194.80967

**Published:** 2022-04-18

**Authors:** Paul M. Peterson, Konstantin Romaschenko, Yolanda Herrera Arrieta

**Affiliations:** 1 Department of Botany MRC-166, National Museum of Natural History, Smithsonian Institution, Washington, DC 20013-7012, USA National Museum of Natural History Washington DC United States of America; 2 Instituto Politécnico Nacional, CIIDIR Unidad Durango-COFAA, Durango, C.P. 34220, Mexico Instituto Politécnico Nacional Durango Mexico

**Keywords:** Classification, *Habrochloa*, molecular phylogenetics, *Nematopoa*, *Neyraudia*, Triraphideae, *Triraphis*

## Abstract

To investigate the evolutionary relationships among species of the tribe Triraphideae (including two monotypic genera, *Habrochloa* and *Nematopoa*), we generated a phylogeny based on DNA sequences from nuclear ribosomal (ITS) and four plastid markers (*rps16-trnK*, *rps16* intron, *rpl32-trnL*, and *ndhA* intron). *Habrochloa* and *Nematopoa* form a clade that is sister to *Neyraudia* and *Triraphis*. Member of the Triraphideae have paniculate inflorescences, 3-veined, marginally ciliate lemmas, usually with hairy lateral veins, that are apically bifid and awned from between a sinus. A description of the Triraphideae and key to the genera is provided, and the biogeography is discussed, likely originating in Africa.

## ﻿Introduction

Clayton and Renvoize (1986) pointed out that *Neyraudia* R. Br. was perhaps an ally of *Triraphis* R. Br. since both genera possess slender microhairs and the two have keeled lemmas that are villous on the lateral veins (Watson and Dallwitz 1992). Based on DNA sequence studies Bouchenak-Khelladi et al. (2008) were first to show strong support for *Neyraudia* and *Triraphis* as being sister in the subfamily Chloridoideae Kunth ex Beilschm. Hilu and Alice (2001) and Bouchenak-Khelladi et al. (2008), using the same *matK* sequence marker placed these two genera in the subtribe Uniolinae Clayton, now a member of tribe Eragrostideae Stapf. Another DNA sequence study supported the placement of the *Neyraudia*–*Triraphis* clade as being sister to remaining species in the Chloridoideae and, subsequently, the tribe Triraphideae P.M. Peterson [based on subtribe TriraphidinaeStapf (1917)] was erected to include these two genera (Peterson et al. 2010). Using unpublished DNA sequence phylogenies (Peterson and Romaschenko, unpubl.), the monotypic *Habrochloa* C.E. Hubb., was added to the Triraphideae in the classification of all genera within the Poaceae (Soreng et al. 2015, 2017).

Hubbard (1935, 1957a, b) transferred *Triraphislongipes* Stapf & C.E. Hubb. to *Crinipes* Hochst. (Arundinoideae) since it possessed a bearded callus, then later moved it to a new monotypic genus, *Nematopoa* C.E. Hubb. *Nematopoa* was included in the Arundinoideae by Clayton and Renvoize (1986). In more recent classifications (Soreng et al. 2015, 2017), *Nematopoalongipes* (Stapf & C.E. Hubb.) C.E. Hubb. was placed as a synonym of *Triraphis* as originally described. Based on unpublished DNA sequence phylogenies (Peterson and Romaschenko, unpubl.), Soreng et al. (2022) and Gallaher et al. (2022) placed *Nematopoa* in the Triraphideae. Therefore, the current concept of the Triraphideae consists of four genera, *Habrochloa*, *Nematopoa*, *Neyraudia*, and *Triraphis*.

*Habrochloabullockii* C.E. Hubb. is a small, delicate, African annual (culms 10–25 cm tall) with a fringe of hairs for a ligule and delicate panicles bearing 3–5-flowered spikelets, each including awned, apically bifid, marginally ciliate lemmas that bear trigonous caryopses, whereas *Nematopoalongipes* is a caespitose, southern African perennial (culms 30–80 cm tall) with ciliate, membranous ligules and capillary panicles bearing 4–7-flowered spikelets, each including awned, apically bifid, marginally ciliate lemmas (Clayton et al. 2016). *Neyraudia* consists of four reedlike perennials [culms (0.8–) 1–5 m tall], a cartilaginous ridge with a line of hairs apically for a ligule, and plumose panicles bearing 3–8-flowered spikelets, each including awned, apically bifid lemmas that are ciliate marginally and along lateral veins; three species in tropical and temperate Asia and one species in Africa (Watson et al. 1992; Filgueiras and Zuloaga 1999; Guala 2003; Clayton et al. 2016). *Triraphis* consists of eight annual or perennials (culms 4−140 cm tall) with membranous ligules or a fringe of hairs and open or contracted (rarely spiciform) panicles bearing 4−24-flowered spikelets, each including apically 3-lobed and 3-awned lemmas that are ciliate marginally and villous along the lateral veins, and trigonous caryopses; six species in Africa, one in Australasia and one in South America (Watson et al. 1992; Nightingale and Weiller 2005; Clayton et al. 2016).

In the present phylogenetic study, using DNA sequences from nuclear ribosomal (ITS) and four plastid markers (*rps16-trnK*, *rps16* intron, *rpl32-trnL*, and *ndhA* intron), we include for the first time *Habrochloabullockii*, *Nematopoalongipes*, and *Neyraudiaarundinacea* (L.) Henrard with two other species of *Neyraudia* and five species of *Triraphis*. In addition, we include a description of the Triraphideae, key to the genera in the tribe, and hypothesize its biogeographical history.

## ﻿Materials and methods

Detailed methods for DNA extraction, amplification, and sequencing are given in Romaschenko et al. (2012) and Peterson et al. (2010, 2014a, b, 2015a, b, 2016). We used Geneious Prime 2020 (Kearse et al. 2012) for contig assembly of bidirectional sequences of *ndhA* intron, *rpl32-trnL*, *rps16* intron, *rps16-trnK* and ITS regions, and implemented in Geneious Muscle algorithm (Edgar 2004) to align the sequences and adjust the ﬁnal alignment. The maximum likelihood parameters for each region were estimated with GARLI 2.0 (Zwickl 2006) and were used as priors in Bayesian calculations to infer overall phylogeny. The Bayesian tree was constructed using MrBayes v3.2.7 (Huelsenbeck and Ronquist 2001; Ronquist et al. 2012). All compatible branches were saved. The Bayesian analysis was initiated with random starting trees sampling once per 100 generations and continued until the value of the standard deviation of split sequences dropped below 0.01 indicating convergence of the chains. The effective sample size (ESS) value for all the parameters was greater than 200 and the first 25% of the sampled values were discarded. Maximum likelihood bootstrap analyses (Felsenstein 1985) were performed using GARLI with 1000 repetitions. The resulted file containing 1000 trees from the bootstrap analysis was then read into PAUP* v.5.0 (Swofford 2000) to compute the majority rule consensus tree.

Our study was designed to test relationships among species residing in four genera (*Habrochloa*, *Nematopoa*, *Neyraudia*, and *Triraphis*) attributed to the Triraphideae. Representative species from all remaining tribes (Centropodieae P.M. Peterson, N.P. Barker & H.P. Linder, Cynodonteae Dumort., Eragrostideae Stapf, and Zoysieae Benth.) in the Chloridoideae have been included to test the monophyly of the tribe (Peterson et al. 2010). In addition, the phylogeny includes two species from the Danthonioideae, *Danthoniacompressa* Austin and *Merxmuelleradrakensbergensis* (Schweick.) Conert, and one species from the Panicoideae, *Chasmanthiumlatifolium* (Michx.) H.O. Yates, which was used as an outgroup.

## ﻿Results and discussion

Thirty-five new sequences (16%) from five species (nine individuals) are newly reported in GenBank, along with all other sequences for 48 individuals and 41 species included in this study (Table 1). Total aligned characters, numbers of sequences, proportion of invariable sites, and other parameters are noted in Table 2. The resulting plastid and ITS topologies were inspected for conflicting nodes with ≥ 95% posterior probabilities. No supported conflict was found so plastid and ITS sequences were combined.

**Table 1.** Taxon voucher (collector, number, and where the specimen is housed), country of origin, and GenBank accession for DNA sequences of *rps16-trnK*, *rps16 intron*, *rpl32-trnL*, *ndhA* intron, and ITS regions; **bold** indicates new accession; a dash (–) indicates missing data, an asterisk (*) indicates sequences not generated in our lab.

	Taxon	Voucher	Country	*rps16-trnK*	*rps16* intron	*rpl32-trnL*	*ndhA* intron	ITS
1	*Centropodiaglauca* (Nees) Cope	Davidse 6367 (US)	South Africa	JF729075	–	JF729175	JF729164	JF729164
2	*Centropodiamossamedensis* (Rendle) Cope	Schweickerdt 2250 (US)	South Africa	JF729076	JF729182	JF729176	–	–
3	*Chasmanthiumlatifolium* (Michx.) H.O. Yates	Peterson 22463 (US)	USA, Maryland	GU360517	GU360438	GU359891	GU359379	GU359319
4	*Chlorisbarbata* Sw.	Peterson 22255& Saarela (US)	Mexico, Sinaloa	GU360514	GU360435	GU359873	GU359377	GU359320
5	*Cotteapappophoroides* Kunth	Peterson 21463, Soreng, LaTorre & Rojas Fox (US)	Peru, Ancash	GU360600	GU360456	GU359842	GU359363	GU359237
6	*Danthoniacompressa* Austin	Peterson 21986 & Levine (US)	USA, North Carolina	GU360521	GU360483	GU359865	GU359370	GU359345
7	*Eleusineindica* (L.) Gaetrn.	Peterson 21362, Saarela & Flores Villegas (US)	Mexico, Mexico	GU360496	GU360472	GU359797	GU359473	GU359338
8	*Eleusinepoiflora* (Chiov.) Chiov.	Burger 2915 (US)	Ethiopia	GU360601	GU360457	GU359843	–	GU359236
9	*Ellisochloarangei* (Pilg.) P.M. Peterson & N.P. Barker	Barker 960 (BOL)	Namibia	JF729079	JF729184	–	JF729166	JQ345167
10	*Enneapogonscaber* Lehm.	Sachse 008 (MO)	South Africa, Western Cape	JQ345237	JQ345279	JQ345322	JQ345208	JQ345168
11	*Entoplocamiaaristulata* (Hack. & Rendle) Stapf	Seydel 187 (US)	South Africa	GU360492	GU360468	GU359793	GU359469	GU359342
12	*Eragrostiskennedyae* F. Turner	Latz 13486 (MO)	Australia	JQ345238	JQ345281	JQ345323	JQ345209	JQ345169
13	*Eragrostiswiseana* (C.A. Gardner & C.E. Hubb.) R.L. Barrett & P.M. Peterson	Peterson 14345, Soreng & Rosenberg (US)	Australia, Western Australia	GU360703	GU360288	GU359986	GU359533	GU359137
14	Gouiniavirgatavar.robusta J.J. Ortíz	Reeder 4714 & Reeder (US)	Mexico, Zacatecas	KF827775	KF827710	KF827639	KF827584	KF827521
15	*Gymnopogongrandiflorus* Roseng., B.R. Arill. & Izag.	Peterson 16642 & Refulio-Rodriguez (US)	Peru, Apurimac	GU360581	GU360383	GU359816	GU359436	GU359200
16	*Habrochloabullockii* C.E. Hubb.	Peterson 23927b, Soreng, Romaschenko & Abeid (US)	Tanzania, Ruvuma	**ON012448**	**ON012442**	**ON012427**	**ON012435**	**OM980631**
17	*Leptocarydionvulpiastrum* (De Not.) Stapf	Peterson 24238, Soreng & Romaschenko (US)	Tanzania	KF827792	KF827725	KF827660	KF827595	KF827539
18	*Leptochloadigitata* (R.Br.) Domin	Risler 476 & Kerrigan (MO)	Australia, Northern Territory	JQ345246	JQ345289	JQ345331	JQ345213	JQ345178
19	*Leptothriumsenegalense* (Kunth) Clayton	Belsky 336 (MO)	Kenya	KF827795	KF827728	KF827663	KF827597	KF827542
20	*Merxmuelleradrakensbergensis* (Schweikerdt) Conert	Mafa 4 (GRA)	South Africa	JF729078	JF729183	–	JF729165	–
21	*Monelytrumluederitzianum* Hack.	Smook 10031 (US)	South Africa	GU360682	GU360421	GU359969	GU359459	GU359158
22	*Mosdenialeptostachys* (Ficalho & Hiern) Clayton	Schweickerdt 1542 (US)	South Africa	GU360681	GU360420	GU359967	GU359458	GU359159
23	*Muhlenbergiajaponica* Steud.	Soreng 5240, Peterson & Sun Hang (US)	China, Yunnan	HM143667	HM143571	HM143183	HM143388	HM143081
24	*Neesiochloabarbata* (Nees) Pilg.	Swallen 4491 (US)	Brazil	GU360724	GU360279	GU360005	–	GU359122
25	*Nematopoalongipes* (Stapf & C.E. Hubb.) C.E. Hubb.	Simon 2353	Africa	MF035992*	MF035992*	MF035992*	MF035992*	–
26	*Neyraudiaarundinacea* (L.) Henrard	Peterson 23991, Soreng, Romaschenko & Abeid (US)	Tanzania, Njomba	**ON012449**	**ON012443**	**ON012428**	**ON012436**	**OM980632**
27	*Neyraudiareynaudiana* (Kunth) Keng ex Hitchc.	Columbus 5302 (RSA)		KF356392*	KF356392*	KF356392*	KF356392*	–
28	*Neyraudiareynaudiana* (Kunth) Keng ex Hitchc.	Soreng 5318, Peterson & Sun Hang (US)	China, Yunnan	–	GU360272	GU360003	GU359397	GU359124
29	*Neyraudiareynaudiana* (Kunth) Keng ex Hitchc.	Srisanga 97923, Norsaengsri, Unwin, Rodda, Schuettpelz, Tin Tin Mu & Ling Shein Man (US)	China, Myanmar	–	–	**ON012429**	–	**OM980633**
30	*Pappophorumpappiferum* (Lam.) Kuntze	Peterson 21689, Soreng, La Torre & Rojas Fox (US)	Peru, Ancash	GU360700	GU360276	GU359996	GU359402	GU359128
31	*Perotisindica* (L.) Kuntze	Peterson 23880, Soreng & Romaschenko (US)	Tanzania	KF827801	KF827734	KF827669	KF827601	KF827546
32	*Psilolemmajaegeri* (Pilg.) S.M. Phillips	Peterson 24247, Soreng & Romaschenko (US)	Tanzania	KM011122	KM010919	KM010695	KM010535	KM010326
33	*Sporobolusvirginicus* (L.) Kunth	Peterson 15683 & Soreng (US)	Chile, Region I	GU360610	GU360362	GU359892	GU359502	GU359215
34	*Tragusberteronianus* Schult.	FLSP 457 (US)	Peru	GU360616	GU360370	GU359898	GU359503	GU359224
35	Tridensflavusvar.chapmanii (Small) Shinners	McCauley 438 (MO)	USA, Missouri	KF827817	KF827751	KF827689	KF827615	KF827568
36	*Triplasisamericana* P. Beauv.	Kral 12065 (MO)	USA, Georgia	KF827818	KF827752	KF827690	KF827616	KJ768887
37	*Triraphisandropogonoides* (Steud.) E. Phillips	Mennell s.n. (US)	South Africa, Cape Province	GU360654	GU360335	GU359949	**ON012437**	–
38	*Triraphismollis* R. Br.	Lazarides 046 & Palmer (US)	Australia, Uluru National Park	–	–	**ON012430**	–	**OM980634**
39	*Triraphismollis* R. Br.	Peterson 14344, Soreng & Rosenberg (US)	Australia, Western Australia	GU360669	GU360336	GU359933	GU359539	GU359187
40	*Triraphismollis* R. Br.	Saarela 1608, Peterson, Soreng & Judziewicz (US)	Australia, Northern Territory	**ON012450**	**ON012444**	**ON012431**	**ON012438**	**OM980635**
41	*Triraphismollis* R. Br.	Saarela 1615, Peterson, Soreng & Judziewicz (US)	Australia, Northern Territory	**ON012451**	**ON012445**	**ON012432**	**ON012439**	**OM980636**
42	*Triraphismollis* R. Br.	Saarela 1648, Peterson, Soreng & Judziewicz (US)	Australia, Northern Territory	**ON012452**	**ON012446**	**ON012433**	**ON012440**	**OM980637**
43	*Triraphismollis* R. Br.	Saarela 1656, Peterson, Soreng & Judziewicz (US)	Australia, Northern Territory	**ON012453**	**ON012447**	**ON012434**	**ON012441**	**OM980638**
44	*Triraphispurpurea* Hack.	Schweickerdt 2115 (US)	Namibia	GU360652	GU360337	GU359932	GU359549	–
45	*Triraphisramosissima* Hack.	Seydel 4278 (US)	Namibia	GU360651	GU360338	GU359931	GU359541	GU359188
46	*Triraphisschinzii* Hack.	Smook 1933 (US)	South Africa	GU360650	GU360339	GU359930	–	–
47	*Uniolacondensata* Hitchc.	Peterson 9342 & Judziewicz (US)	Ecuador, Chimborazo	GU360649	GU360340	GU359927	GU359534	GU359191
48	Zoysiamacranthasubsp.walshii M.E. Nightingale	Loch 435 (US)	Australia	GU360642	GU360345	GU359922	GU359548	GU359197

**Table 2.** Characteristics of the five DNA regions (*rps16-trnK*, *rps16* intron, *rpl32-trnL*, *ndhA* and ITS) and parameters used as priors in Bayesian analyses estimated with GARLI. 2.0.

Characteristic	*rps16-trnK*	*rps16* intron	*rpl32-trnL*	*ndhA* intron	Combined plastid data	ITS	Overall
Total aligned characters	887	1046	844	1146	3923	769	4692
Number of sequences	45	45	46	42	178	41	219
Number of new sequences	6 (13%)	6 (13%)	8 (17%)	7 (17%)	27 (15%)	8 (20%)	35 (16%)
Likelihood score (-lnL)	3909.0	3405.6	3778.7	4281.4		7973.0	
Number of substitution types	6	6	6			6	
Model for among-sites rate variation	gamma	Gamma	gamma			gamma	
Substitution rates	1.2071 2.7093 0.4083 1.5405 2.9778 1.0000	1.2951 1.2876 0.3028 1.1547 2.0746 1.0000	1.0625 1.7914 0.3251 1.4401 1.5146 1.0000	0.9848 2.5216 0.2912 1.9389 2.3679 1.0000	–	1.1422 2.6273 1.7222 0.6568 4.5253 1.0000	
Character state frequencies	0.3088 0.1363 0.1462 0.4084	0.3779 0.1226 0.1743 0.3251	0.3693 0.1380 0.1222 0.3703	0.3669 0.1348 0.1484 0.3497	–	0.2404 0.2374 0.2582 0.2641	
Proportion of invariable sites	0.1666	0.3154	0.0413	0.2537	–	0.2547	
Gamma shape parameter (α)	2.1848	1.0833	0.9498	1.0636	–	0.9409	

**Figure 1.**
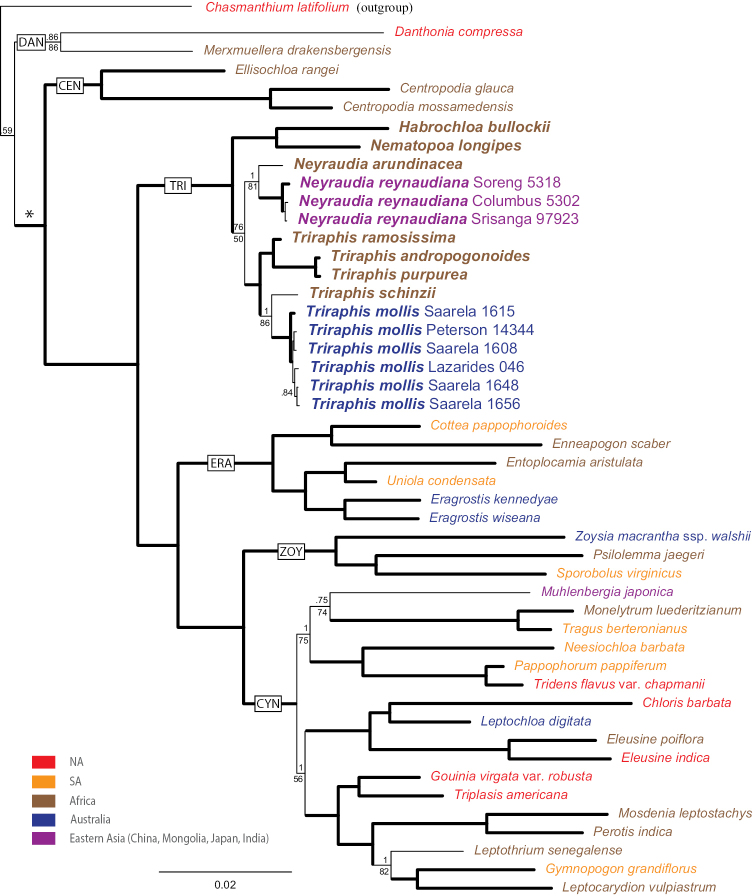
Maximum-likelihood tree inferred from combined plastid (*rps16-trnK*, *rps16* intron, *rpl32-trnL*, and *ndhA* intron) and ITS sequences. Numbers above branches are posterior probabilities; numbers below branches are bootstrap values; thick branches indicate bootstrap ≥ 90% and posterior probabilities of 1.00; DAN = Danthonioideae; tribes within the Chloridoideae = *, include: CEN = Centropodieae, TRI = Triraphideae, ERA = Eragrostideae, ZOY = Zoysieae, and CYN = Cynodonteae. Scale bar: 2%.

The Bayesian tree from the combined plastid and ITS regions is well resolved (Fig. 1). Most clades have posterior probabilities equal to 1.00 and additional bootstrap values of 90% or greater. There is strong support for *Habrochloabullockii* + *Nematopoalongipes* sister to a monophyletic *Neyraudia* with three individuals of *N.reynaudiana* (Kunth) Keng ex Hitchc. sister to one individual of *N.arundinacea* (type of the genus) plus a monophyletic *Triraphis*. The *Triraphis* clade includes six individuals of *T.mollis* R. Br. (type of the genus as treated by Burbidge 1946 and Peterson et al. 2022) sister to *T.schinzii* Hack. and *T.ramosissima* Hack. sister to *T.andropogonoides* (Steud.) E. Phillips + *T.purpurea* Hack. Our molecular data clearly support independent recognition of *Nematopoa* since it is sister to *Habrochloa* and not a member of the *Triraphis* clade.

*Habrochloabullockii* and *Nematopoalongipes* are clearly aligned within the Triraphideae, and together with *Neyraudia* and *Triraphis*, share the following salient morphological features: paniculate inflorescences, 3-veined, marginally ciliate lemmas, usually with hairy lateral veins, and lemmas that are apically bifid and awned from between the sinus (Watson and Dallwitz 1992; Watson et al. 1992; Peterson et al. 2010; Clayton et al. 2016). Another probable synapomorphy for these four genera is possession of panicoid-type bicellular microhairs (long, narrow basal and terminal cells; Amarasinghe and Watson 1988). Watson et al. (1992) verified the presence of panicoid bicellular microhairs for *Habrochloa*, *Nematopoa*, and *Triraphis* but indicate that they are absent in *Neyraudiaarundinacea*. However, Clayton and Renvoize (1986) previously indicated that *Neyraudia* possesses slender microhairs similar to those in *Triraphis*.

Based on a sample containing *Nematopoa*, *Neyraudia*, and *Triraphis*, Gallaher et al. (2022) determined the crown age (10.62 Ma) and stem age (46.76 Ma) of the Triraphideae. Although at least three species of *Neyraudia* include tropical and temperate Asia in their distribution, Africa is the most likely area of origin for the Triraphideae since all four genera in the tribe include species distributed in Africa. In addition, the Triraphideae shares a common ancestor with Centropodieae, also from Africa and temperate Asia (Peterson et al. 2011). Because more than half of the genera of Chloridoideae reside in Africa and the larger tribes, i.e., the Eragrostideae and Zoysieae have centers of diversity there, Hartley and Slater (1960) earlier concluded that the subfamily probably originated on the African continent and spread to other parts of the world (Bouchenak-Khelladi et al. 2008; Peterson et al. 2007, 2010, 2011, 2014c).

## ﻿Taxonomy

### ﻿ Triraphideae

Taxon classificationPlantaePoalesPoaceae

P.M. Peterson, Molec. Phylogen. Evol. 55(2): 591. 2010 ≡ Triraphidinae Stapf, Fl. Trop. Afr. 9: 22. 1917 – Type: Triraphis R. Br., Prodr. 185. 1810.

FE38B36E-D39D-5DBA-9683-72C7CC8253A7

#### Description

**(emendation).** Annuals or perennials, sometimes rhizomatous or reedlike (*Neyraudia*) culms 4–500 cm tall, erect or decumbent; ligules membranous and ciliate or a fringe of hairs; inflorescence a panicle, open to contracted, rarely spiciform; spikelets 2–15 mm long, 3–24-flowered, laterally compressed; glumes usually shorter than the spikelets or upper glume 2 × as long as adjacent lemma (*Habrochloa*), 0-, 1- or 3-veined, membranous, sometimes hyaline, apex entire to mucronate, rarely awned; lemmas membranous, rarely cartilaginous, 3-veined with ciliate or pilose margins, lateral veins, if present, usually hairy and sometimes extending as awns (*Triraphis*), apex bifid and awned from between the sinus; paleas 0.5 to as long as lemma, 2-veined; stamens 3; caryopses with adherent pericarp, often trigonous to ellipsoid, sometimes linear.

#### Included genera.

*Habrochloa*, *Nematopoa*, *Neyraudia*, *Triraphis*.

### ﻿Key to the genera

**Table:** 

1	Lemmas 3-awned, the lateral veins extending into awns	***Triraphis***
–	Lemmas 1-awned, the lateral veins never extending into awns	**2**
2	Culms (80–) 100–500 cm tall, generally 1–1.5 cm wide at base, often woody; plants perennial, reedlike; ligules cartilaginous at base, apically with a line of hairs; panicles 30–80 cm long, plumose	***Neyraudia***
–	Culms 10–80 cm tall, ≤ 3 mm wide at base, herbaceous; plants annual not reedlike; ligules membranous with a fringe of hairs, not cartilaginous at base; panicles 2–30 cm long, not plumose	**3**
3	Spikelets 2–2.5 mm long; lemmas 1–1.3 mm long, 3-veined, awned, the awns 4–6 mm long; upper glumes 2 × as long as adjacent lemma	***Habrochloa***
–	Spikelets 6–10 mm long; lemmas 3.5–4.3 mm long, 1-veined, awned, the awns 8–13 mm long; upper glumes 0.5–0.6 × as long as adjacent lemma	***Nematopoa***

## Supplementary Material

XML Treatment for Triraphideae
